# TrueAllele Casework on Virginia DNA Mixture Evidence: Computer and Manual Interpretation in 72 Reported Criminal Cases

**DOI:** 10.1371/journal.pone.0092837

**Published:** 2014-03-25

**Authors:** Mark W. Perlin, Kiersten Dormer, Jennifer Hornyak, Lisa Schiermeier-Wood, Susan Greenspoon

**Affiliations:** 1 Cybergenetics, Pittsburgh, Pennsylvania, United States of America; 2 Department of Forensic Science, Richmond, Virginia, United States of America; University of Central Florida, United States of America

## Abstract

Mixtures are a commonly encountered form of biological evidence that contain DNA from two or more contributors. Laboratory analysis of mixtures produces data signals that usually cannot be separated into distinct contributor genotypes. Computer modeling can resolve the genotypes up to probability, reflecting the uncertainty inherent in the data. Human analysts address the problem by simplifying the quantitative data in a threshold process that discards considerable identification information. Elevated stochastic threshold levels potentially discard more information. This study examines three different mixture interpretation methods. In 72 criminal cases, 111 genotype comparisons were made between 92 mixture items and relevant reference samples. TrueAllele computer modeling was done on all the evidence samples, and documented in DNA match reports that were provided as evidence for each case. Threshold-based Combined Probability of Inclusion (CPI) and stochastically modified CPI (mCPI) analyses were performed as well. TrueAllele’s identification information in 101 positive matches was used to assess the reliability of its modeling approach. Comparison was made with 81 CPI and 53 mCPI DNA match statistics that were manually derived from the same data. There were statistically significant differences between the DNA interpretation methods. TrueAllele gave an average match statistic of 113 billion, CPI averaged 6.68 million, and mCPI averaged 140. The computer was highly specific, with a false positive rate under 0.005%. The modeling approach was precise, having a factor of two within-group standard deviation. TrueAllele accuracy was indicated by having uniformly distributed match statistics over the data set. The computer could make genotype comparisons that were impossible or impractical using manual methods. TrueAllele computer interpretation of DNA mixture evidence is sensitive, specific, precise, accurate and more informative than manual interpretation alternatives. It can determine DNA match statistics when threshold-based methods cannot. Improved forensic science computation can affect criminal cases by providing reliable scientific evidence.

## Introduction

DNA analysis is the forensic gold standard in human identification [1]. By deriving a genotype from minute amounts of biological material [2], scientists can help identify individuals connected to a crime scene.

With increased societal expectations [3], crime laboratories now process more challenging DNA evidence. Such samples are typically mixtures of two or more individuals, with DNA that may be damaged, degraded or present in small amounts [4]. DNA from one person expresses only one or two alleles at a genetic locus, and so is readily genotyped by visual inspection. Mixture data, however, may present multiple genotype alternatives that complicate interpretation.

Human analysts may simplify short tandem repeat (STR) [5] interpretation by applying a threshold that reduces quantitative data into all-or-none events [6]. This approach works well with single source samples that contain only one genotype. But with mixtures, thresholds discard the quantitative contributions of each genotype, along with the peak height pattern. Threshold-based methods can reduce identification information, render probative data “inconclusive”, and potentially infer an incorrect genotype [7].

An “analytical” threshold helps human analysts distinguish between allelic data peaks and baseline instrument noise. The Combined Probability of Inclusion (CPI) mixture interpretation method first applies this analytical threshold to decide which peaks at a locus are sufficiently tall to be considered alleles. If a reference individual’s alleles are included in this set of mixture alleles, then CPI uses all the alleles in the mixture set to calculate a match statistic (the inclusion probability) as the square of the sum of the allele frequencies. (Allele determination can be viewed as a separate human interpretation step that precedes the CPI statistical calculation step. For clarity in this paper, we consider the entire data analysis procedure to comprise the CPI interpretation method). The method does not make assumptions about the number of contributors.

There is naturally occurring random variation in the polymerase chain reaction (PCR) [8]. Therefore, repeat amplifications of the same DNA template quantity will produce varying peak heights. A pair of heterozygote sister alleles may express one taller peak, along with a considerably shorter peak, thereby creating a situation where the heterozygote could be misinterpreted as a homozygote. The analytical threshold does not address this situation [9].

In 2010, the United States Scientific Working Group on DNA Analysis Methods (SWGDAM) published guidelines to help resolve such mixture genotyping issues [10]. For manual mixture review, these new SWGDAM guidelines introduced a higher “stochastic” threshold for use in a modified CPI (mCPI) mixture interpretation method. After determining locus alleles using the analytical threshold, and establishing that an individual is included, the more stringent mCPI method additionally requires that every allele over the analytical threshold must also reach the stochastic threshold; otherwise the locus cannot be used in the mCPI match statistic. The taller peak height requirement addresses genotype errors by statistically removing ambiguous locus situations where a peak resides in a third state, between the analytical and stochastic thresholds. However, mCPI can discard potentially useful identification data, which lowers match statistics and reclassifies previously interpretable mixtures as “inconclusive”.

The Virginia Department of Forensic Science (DFS) implemented the new SWGDAM mixture interpretation guidelines, and reviewed their DNA evidence using stochastic thresholds. In 2011, DFS identified 375 criminal cases in which their stochastic threshold method had produced an inconclusive result or a less informative match statistic [11]. Interested in preserving more identification information, DFS employed a provision in the SWGDAM guidelines (paragraph 3.2.2) that allowed use of a validated “probabilistic genotyping” computer interpretation method [10].

Mathematical modeling can account for quantitative STR data patterns [12]. Combining different amounts of contributor genotypes, along with other variables, produces allele patterns that can be compared with STR data peaks [13]. Incorporating probability into the equations allows a computer to assess the relative likelihood of alternative solutions [14], [15]. The result is a genotype probability distribution that is objectively derived from the data, independent of known comparison genotypes. Subsequent comparison of this evidence genotype with a reference genotype, relative to a human population, produces a DNA match statistic that measures identification information. By using all of the quantitative DNA mixture data, and thoroughly considering all feasible genotype alternatives, computer modeling can preserve more identification information than manual review [7].

DFS pursued a probabilistic genotyping approach for their DNA mixture evidence. They arranged for Cybergenetics (Pittsburgh, PA) to apply their validated TrueAllele Casework system to DNA mixture evidence in 144 cases. Cybergenetics produced DNA match reports on 92 evidence items in 72 cases. This is the largest data set on which case reports have been generated for probabilistic genotyping of DNA mixture evidence.

This study describes the results of computer-based probabilistic genotyping mixture interpretation on 101 reported matches, out of 111 genotype comparisons. (A DNA match is defined here operationally as a comparison between an evidence and reference genotype, relative to a population, that gives a reproducible positive match statistic). The 10 comparisons that did not produce a match are also characterized. The study compares the computer’s information yield with two methods of manual interpretation on the same evidence items. Previous TrueAllele Casework validation studies have been published on samples of known composition [13], [16], as well as on actual casework items [7], [17]. This observational study was performed on casework items. A companion study has been performed using laboratory synthesized mixtures [18].

The present study compares three interpretation methods for analyzing DNA mixtures from actual casework, specifically, the automated TrueAllele Casework computer system, with the traditional CPI and updated mCPI manual threshold-based methods [19]. The manual methods involve determining and applying a threshold for binary or ternary peak classification, whereas the automated approach uses continuous peak quantities without a threshold (Table 1). The study hypothesizes that the automated system will be more statistically powerful, precisely because it uses more of the data that manual methods discard. If this hypothesis is correct, the automated system should generally reach the same conclusions but infer more powerful match statistics, and resolve cases that the manual methods do not.

**Table 1:** Distinguishing features of three different DNA mixture interpretation methods.

		TrueAllele	CPI	mCPI
**Peak data**	*Approach*	quantitative	qualitative	qualitative
	*Scale*	continuous	binary	ternary
	*Height*	used		
	*Pattern*	used		
	*Threshold*		analytical	analytical and stochastic
**Genotype**	*Inference*	probability model	data above analyticalthreshold	data above analyticalthreshold
	*Representation*	allele pairs	alleles	alleles
	*Operation*	automated	manual	manual
	*Inclusion*	statistical	alleles	alleles
	*Contributor number*	assumed		
**Statistic**	*Comparison*	with genotype	with alleles	with alleles
	*Locus*	all	inclusion	stochastic inclusion
	*Calculation*	likelihood ratio	inclusion probability	inclusion probability
	*Application*	include, exclude orinconclusive	include	include
	*Identification*	information	inclusion	inclusion

Attributes involving STR data usage, genotype inference and match statistic calculation are shown for the TrueAllele, CPI and mCPI methods.

The paper begins by describing three methods of DNA mixture interpretation, one automated and two done manually. We then present the case materials used and the interpretation procedures employed. We examine the TrueAllele automation method’s reliability, using its inferred match statistics to assess how sensitive, specific, precise and accurate it is. (In Forensic Science, “sensitive” and “specific” describe the reliability of analytical instrumentation. With DNA interpretation methods, they can similarly describe the respective degree of positive or negative identification). We compare how well TrueAllele, CPI and mCPI preserve identification information relative to one another, as measured by their match statistics. We conclude by observing that computer-based DNA mixture interpretation can provide an improvement over current manual forensic processes.

## Methods

The DNA samples used in this study were lawfully obtained by DFS in accordance with Virginia code Section 9.1–1101. All personal identifiers were removed from DNA data prior to computer interpretation. The submitted scientific manuscript contains only summary statistics, and discloses no personal or case information.

The DNA mixture interpretation process begins with electronic data signals. These signals are examined to form genotypes. Comparison of an evidence genotype with a reference genotype, relative to a population, can then produce a DNA match statistic.

### STR Mixture Data

A STR locus is a length polymorphism, where alleles have different numbers of short DNA units (typically four or five base pairs) that are repeated in tandem [5]. When a polymorphic locus has 15 or more alleles, it provides over a hundred possible genotype values. This genetic variation is useful for distinguishing between people in a population. For example, the Penta E locus on chromosome 15 contains the five base pair repeat unit (AAAGA)_n_, with n = 5, 6, …, 24; these 20 alleles permit 210 distinct allele pairs. (Given n alleles, there are n(n+1)/2 possible unordered allele pairings, with n homozygotes and n(n–1)/2 heterozygotes. With n = 20, there are 20⋅21/2, or 210 genotype values).

Following DNA extraction and quantification, STR analysis proceeds in two steps. First, PCR amplification with a set of fluorescently labeled primers creates millions of allele copies from the DNA template. Random variation in a 31 cycle PCR process [19] produces natural variation in the quantities of amplified alleles [20]. Second, the allele amplicons are size-separated by capillary electrophoresis, with laser detection of DNA quantity measured in relative fluorescent units (RFU). The amplified allele size and quantity signals are recorded as peaks in an electropherogram (EPG), and saved into a fragment size analysis (.fsa) data file.

**Figure 1:**
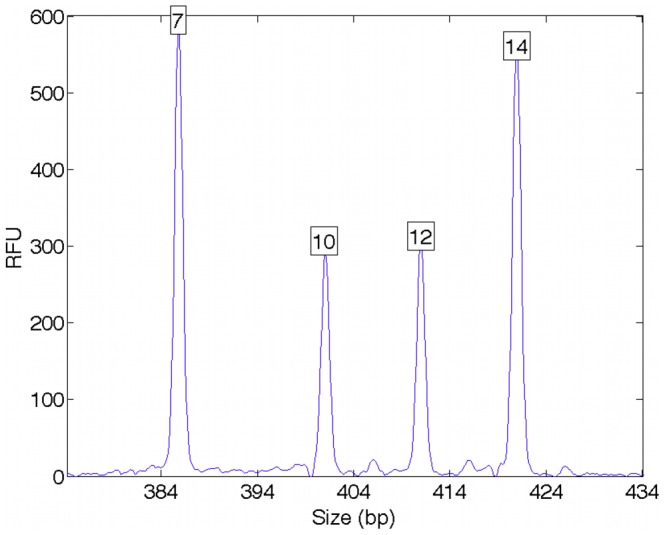
Mixture data. Quantitative DNA mixture data are shown at the Penta E STR locus. The x-axis measures allele fragment size (bp), and the y-axis measures DNA quantity (RFU); a boxed peak number denotes allele length. The two contributor mixture is formed from a 7,14 major genotype and a 10,12 minor genotype. The result is a pattern of peak heights that reflect the underlying genotypes.

Penta E is one of 15 STR loci in the Promega PowerPlex 16 multiplex kit [21]. The example EPG data at this locus show a pattern of allelic peaks, where the x-axis (molecular size) corresponds to the allele’s number of repeats and the y-axis (RFU height) relates to allele quantity (Figure 1). The data have two tall peaks for alleles 7 and 14 with heights around 600 RFU, and two shorter peaks at alleles 10 and 12 of height 300 RFU.

### Three Interpretation Methods

STR mixture data can be interpreted in different ways, giving rise to different DNA match statistics. Table 1 lists the features of three such methods – the quantitative computer-based TrueAllele approach, as well as the two qualitative human review methods CPI and mCPI. TrueAllele uses all the peak height data on a continuous RFU scale, examining the entire peak pattern to make inferences. Applying an analytical RFU threshold, CPI reduces the peak height quantities to two binary states (allele or not), while mCPI additionally applies a higher stochastic threshold to develop a third state (uncertain).

TrueAllele infers genotypes with a probability model that uses a computer to automatically propose peak patterns, and assess how well they explain the quantitative data (Table 1, Genotype). The manual methods infer alleles based on events above a predetermined analytical threshold RFU level, and then assess inclusion. Because TrueAllele separates out the genotypes contributing to a mixture, it can compare evidence genotypes (as probability distributions) with reference genotypes. CPI and mCPI reduce peak height data to “alleles” instead of separating out genotypes, and so compare reference genotypes with evidence data features instead of with inferred genotypes.

TrueAllele’s inferred (probabilistic) genotypes can be entered into standard formulae to calculate a likelihood ratio (LR) (Table 1, Statistic). This LR result can give weight to inclusion or exclusion, and so all loci are used in the match statistic [22]. CPI and mCPI first establish an inclusion based on the analytical threshold; loci that do not support an inclusion are not assigned a probability of inclusion. The mCPI statistical calculation will not use a locus that has an uncertain allele whose peak height lies between the analytical and stochastic thresholds.

### TrueAllele Genotype Modeling

Many variables are considered in genotype modeling, such as the genotypes of each contributor at every locus, the mixture weights (that sum to 1) of the contributors, the DNA template mass, PCR stutter, relative amplification, DNA degradation and the uncertainties of all these variables. A likelihood function assesses how well particular values of these variables explain the observed quantitative STR data peaks, determining the probability of the (fixed) data conditioned on the (changing) variable values.

With DNA mixture data vector **d** (of peak heights and sizes) having K contributors, the primary explanatory variables are the genotypes *G*, mixture weight *W* and mass *M* (of combined allelic fluorescence intensity). An approximate likelihood function containing these variables is
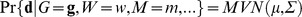
where mean pattern vector 
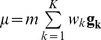
 and covariance matrix 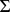 are parameters of a multivariate normal (*MVN*) distribution, as previously described [7], [13]. Pattern 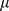 is constructed as a weighted sum of contributor allele pairs **g_k_**.

**Figure 2:**
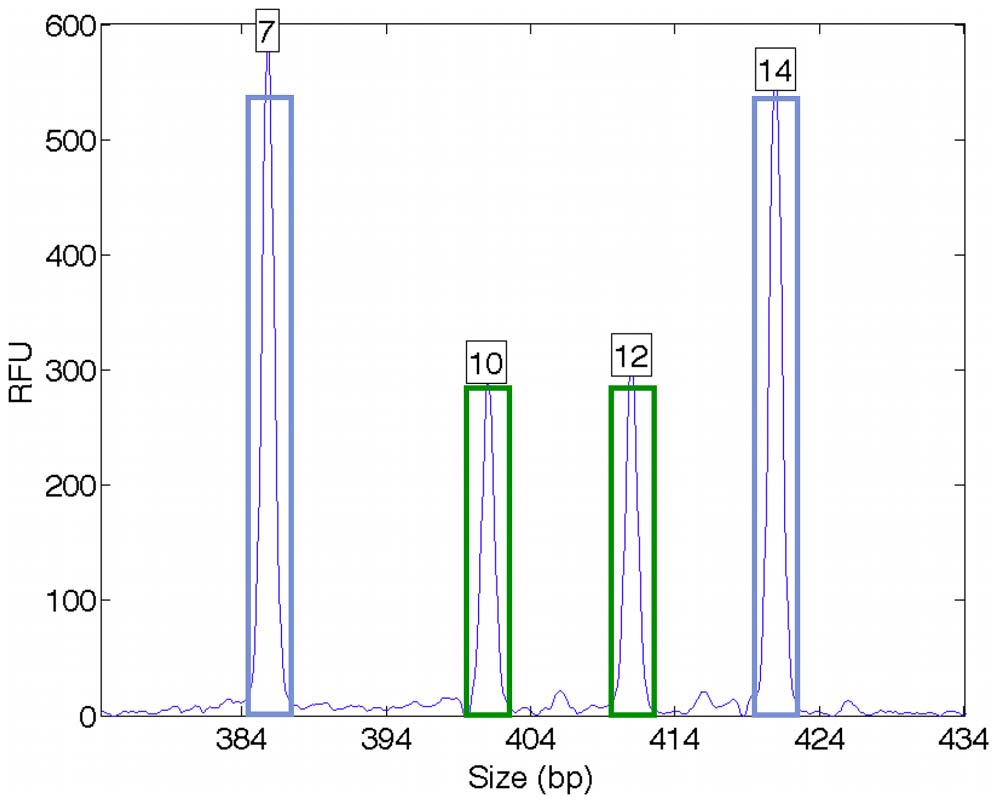
Genotype modeling. Linear combinations of genotype allele pairs can explain the observed quantitative mixture data. Here, a major 7,14 contributor (blue bars) having twice the DNA as a minor 10,12 contributor (green bars) explains the data well, with a high likelihood value. Alternative genotype choices or combinations would not explain the data as well, and thus have lower likelihood.

We can visually understand this likelihood function as constructing a pattern of allele heights that can be compared with the peak height data. Figure 2 shows a major contributor 7,14 allele pair (blue rectangles of equal height) and a minor 10,12 genotype value (green rectangles of equal height) in a 2∶1 mixture ratio. This genotype model is superimposed on the STR peak data, where we see a good fit between the model and data patterns, which corresponds to a high likelihood value. Alternative genotype values and amounts might not explain the data as well (e.g., proposing genotypes 7,10 and 12,15), and would have a lower likelihood.

The posterior genotype probability is proportional to the likelihood value times the prior population probability [23]

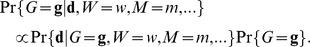



Bayes theorem [24] requires us to consider all feasible genotype alternatives, even those having little probability. Other variables, such as mixture weight, are similarly framed as posterior probability distributions [7]. Since the high dimensional parameter space is vast, the TrueAllele computer conducts a statistical search using Markov chain Monte Carlo (MCMC) to thoroughly sample the joint posterior probability distribution [25], [26].

The modeling approach is objective in the sense that only evidence data is used to infer genotypes, without any knowledge of a reference comparison genotype. Proceeding *ab initio* from the data and model eliminates natural examination bias issues that may affect other mixture interpretation approaches [27]. The resulting evidence genotype for the minor contributor at this locus concentrated 98% of its probability at allele pair 10,12 (Table 2, TrueAllele).

**Table 2:** Genotype probabilities and LR calculations are shown at the Penta E locus for a minor contributor.

Allele Pair			TrueAllele			CPI			mCPI		
		prior	likelihood	posterior	LR	likelihood	posterior	LR	likelihood	posterior	LR
7	7	4.3%				1	17%		1	67%	
7	10	3.3%	2			1	13%				
7	12	7.1%	2	1%		1	28%				
7	14	1.9%				1	8%		1	30%	
10	10	0.6%	1			1	2%				
*10*	*12*	*2.7%*	*986*	*98%*	*37*	*1*	*11%*	*4*			*0*
10	14	0.7%				1	3%				
12	12	2.9%	8	1%		1	11%				
12	14	1.6%	1			1	6%				
14	14	0.2%				1	1%		1	3%	
Total				100%			100%			100%	

Three different mixture interpretation methods were used, TrueAllele, CPI and mCPI. Over the sample space of possible allele pairs, each method has a likelihood function and posterior probability distribution. The LR gives the ratio of posterior to prior probability at comparison allele pair 10,12 (italicized row). TrueAllele’s greater LR indicates more use of the STR data than CPI. mCPI discarded too much data, and could not yield a match statistic.

### CPI Allele Inclusion

**Figure 3:**
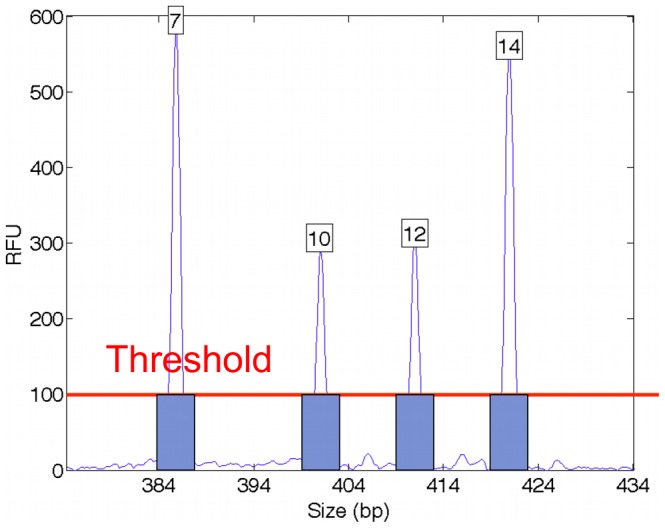
Analytical threshold. The purpose of this threshold is to distinguish allelic signal from background noise. Applying the threshold (red line) reduces the quantitative peaks to all-or-none putative allele events (blue bars). The analytical threshold operation eliminates individual peak heights, as well as their collective pattern.

Inclusion methods of DNA mixture interpretation begin by applying an analytical threshold to the quantitative STR peak data. The Virginia DFS analytical thresholds are specific to each fluorescent dye channel: 73 RFU (blue dye), 84 RFU (green), 75 RFU (yellow) and 52 RFU (red). Peaks above the threshold are designated as “allele” events, while those below are not used (Figure 3).

The inclusion likelihood function assigns 1 to all allele pairs included in the allele list, and 0 otherwise. This CPI likelihood also assumes that all alleles from each contributor are present. With four allele events, for example, there are ten possible allele pairs (Table 2, CPI). Multiplying the prior probability times the 0/1 likelihood values, and renormalizing, gives the CPI genotype probability distribution [28].

The inclusion approach disperses probability over (in this example) ten genotype values. Many of these allele pairs (e.g., 7,7) are not compatible with a minor contributor genotype, based on the peak height data shown. Since the total probability is 1, diverting genotype probability away to infeasible solutions reduces the probability at more likely solutions, and thereby lowers match strength. Starting from highly informative STR data, CPI may reduce considerably the reported identification information, or even eliminate it entirely by dismissing an evidence item as “inconclusive”. Inclusion protocols are susceptible to examination bias, since a reference genotype could be considered (e.g., to assess potential allelic dropout) when determining whether to use a locus in a CPI statistical calculation [29].

### mCPI Stochastic Threshold

Replicate STR experiments exhibit natural variation in peak height, as described by probability model data variance parameters [13], [30]. When interpreting DNA evidence using threshold approaches, stochastic thresholds help to account for this peak pattern variability, which is often more pronounced in low-template samples [8]. A laboratory determines its stochastic threshold through replicate PCR experiments that examine heterozygote allele imbalance and drop out. For example, in following the SWGDAM 2010 guidelines, Virginia DFS set its stochastic thresholds for different capillary injection times as 210 RFU (2s), 320 RFU (5s) and 460 RFU (10s) [19].

**Figure 4:**
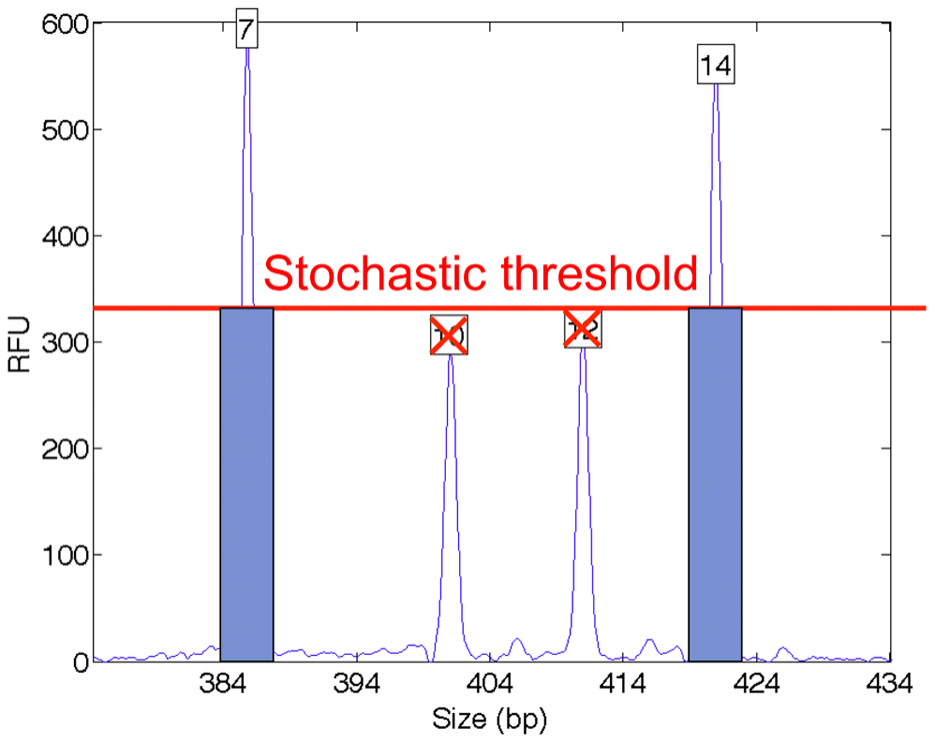
Stochastic threshold. A higher threshold level (red line) is used in manual review to address random peak variation by differentiating more certain (blue bars) from less certain peaks. The stochastic threshold removes more STR loci from statistical consideration, which makes less use of the available data.

The higher mCPI stochastic threshold can make less use of the STR data. In our Penta E mixture example, the 5s injection peak heights of alleles 10 and 12 now fall below the stochastic threshold of 320 RFU (Figure 4). This peak removal can assign essentially zero probability to a 10,12 minor contributor allele pair at this locus in a statistical calculation (Table 2, mCPI). Manual mCPI mixture interpretation would omit Penta E from the cumulative match statistic because of the uncertain alleles 10 and 12, and thus not report the identification information at that locus.

### Likelihood Ratio

The likelihood ratio is a standard DNA match statistic [31]. The LR summarizes in one number the impact of STR data on our belief in the identification hypothesis H that an individual contributed their DNA to biological evidence. The base ten logarithm of the LR expresses identification information in additive “ban” units, and is called the “weight of evidence” [32].

There are several ways to calculate a LR match result, all of which produce the same number [33]. Since our focus here is on genotypes, we note that the LR is the ratio of posterior (after having seen evidence) to prior (the population distribution) genotype probabilities, evaluated at the allele pair of a known reference [34]. For case reporting, we write “a match between the evidence and reference is (some number) times more probable than coincidence”. The LR can also account for co-ancestry, the relatedness in populations between all people [35], [36].

When a genotype likelihood function accounts for observed quantitative evidence data, a reproducibly inferred LR number can accurately summarize the extent of match between that evidence and a reference. A positive log(LR) provides a weight of evidence supporting a match, a negative log(LR) does not favor a match, while a log(LR) near zero is inconclusive. The LR value is always scientifically meaningful. Scientists sometimes verbally describe a LR using an arbitrary subjective scale [37].

### Comparing Three Interpretation Methods

Table 2 shows the prior, likelihood and posterior genotype probabilities of a minor contributor for three different mixture interpretation methods at locus Penta E. The prior probability is the population prevalence of an allele pair (Table 2, prior). The differences between these methods reside in their likelihood functions (Table 2, likelihood):

- The *TrueAllele genotype modeling* likelihood function is a positive real number that describes how well each allele pair hypothesis explains the STR data.
- With *CPI allele inclusion*, the analytical threshold produces a list of included possible allele pairs; these receive a likelihood of 1, and all other values are set to 0.
- Using a *mCPI stochastic threshold* at a higher RFU level forms a possibly shorter list of allele pairs, corresponding to binary 0/1 likelihoods.

A method’s posterior genotype probability is the product of its likelihood and the prior, normalized to sum to unity (Table 2, posterior).

The LR is shown (Table 2, LR) as a ratio of posterior to prior genotype probabilities. We see that TrueAllele genotype modeling used peak height information to make a clear distinction between the 7,14 major and 10,12 minor genotype contributors. By ascribing 98% of the probability to genotype 10,12, the continuous computer method produced a LR of 37 (posterior to prior ratio of 98/2.7) that preserved virtually all of the identification information.

The CPI allele inclusion method uses all data peaks above a predetermined analytical threshold to form allele pairs [38]. The LR of the inclusion genotype at known 10,12 relative to the population is 4 (11/2.7), the reciprocal of the inclusion probability. The inclusion method’s LR of 4 at this locus is approximately an order of magnitude less than TrueAllele’s genotype modeling LR of 37. Multiplying together independent locus inclusion LR values gives the CPI match statistic. The inclusion method is named CPI by its match statistic, also dubbed Random Man Not Excluded (RMNE).

mCPI uses a stochastic threshold to produce a DNA match statistic. mCPI only uses those loci at which all of the peaks are above the stochastic threshold. In our Penta E locus example, the data peaks corresponding to the known 10,12 individual are both under the threshold, setting the mCPI likelihood to zero. Therefore the mCPI posterior probability and LR (from the calculation 0/2.7) of the locus would both be zero, as well. This locus was not used in the mCPI calculation.

Considering all loci in this mixture, TrueAllele’s log(LR) was 16.32; the weight of evidence was 7.04 ban for CPI, and 6.00 ban for mCPI. This example illustrates how genotype modeling makes more use of the data to preserve DNA match information, while an already diminished CPI match statistic can be further reduced by the mCPI stochastic threshold. Our study examines this phenomenon on a larger set of Virginia DFS case matches, comparing the three mixture interpretation methods TrueAllele, CPI and mCPI.

## Materials

### Mixture Data

The Virginia DFS identified DNA mixture cases where computer interpretation could potentially make more use of the STR data than manual review. The selection criteria included having a probative DNA item, possible use of that item as evidence in a criminal trial, an included person of interest, and a need for accurate DNA match information. Items that were easy to interpret manually were not chosen.

The 72 cases spanned a full range of biological evidence, including touch, epithelial cells, blood, saliva and semen (Table 3). These samples are representative of DNA laboratory casework items. The DNA evidence items were all mixtures, most having 3 contributors and some 4 (Table 4), as estimated visually from locus peak counts and patterns.

**Table 3:** The range of biological sample types that were found in the 92 evidence items is shown.

Sample type	Count
blood	10
epithelial/skin	30
fingernails	2
hair	1
saliva	4
semen	3
stain	1
touch	41

For each sample type, the table records how frequently that type was seen.

**Table 4:** The first three rows estimate for each number of contributors (first column) how many mixture items (second column) had that contributor number.

	Contributors	Items
**Estimate**	2	40
	3	65
	4	8
**Overlap**	2 or 3	16
	3 or 4	3
	2, 3 or 4	1

When an item was consistent with more than one contributor number possibility, that item appears in multiple categories. The last three rows examine overlap situations where the number of contributors (first column) was uncertain, and counts the number of items (second column) in those situations.

The mixture weights as calculated by TrueAllele were evenly distributed between 10% and 90% (Table 5). Statistically comparing this empirical mixture weight distribution with a uniform probability distribution gave a Kolmogorov-Smirnov test [39] statistic of 0.1079, whose p-value (0.2220>0.05) showed no significant difference between the distributions.

**Table 5:** The frequency distribution of mixture weights as inferred by the computer is shown for the matched genotypes.

Mixture Weight	Count
0.05	3
0.15	13
0.25	5
0.35	12
0.45	18
0.55	12
0.65	11
0.75	12
0.85	12
0.95	4

The binning is done by decile, with each row showing the center of its mixture weight range, along with the number of genotypes in that bin.

Virginia DFS generated STR data using the Promega PowerPlex 16 kit (Madison, WI), analyzed on an Applied Biosystems 3130×l Genetic Analyzer (Foster City, CA). The DFS case materials included electronic.fsa data files from the sequencer, their own case reports and case context descriptions. DFS sent these electronic materials via secure file transfer protocol (sFTP) to Cybergenetics during the latter half of 2011. The data files were organized in batches for computer processing.

### TrueAllele System

Cybergenetics TrueAllele Casework is a computer system for resolving DNA mixtures into their component genotypes [40]. Written in the MATLAB programming language, the computer uses MCMC sampling [25] to solve a hierarchical probability model [23]. (In this paper, a “computer” always refers to TrueAllele Casework software running on either a client or server computer). A human operator uses VUIer (Visual User Interface) client software that interfaces over a network with a server that hosts a database and parallel processors running interpretation software.

TrueAllele divides DNA identification into two phases [41]. The computer first infers genotypes from the evidence data. The inference is objective in that it has no knowledge of downstream comparison reference genotypes. Afterwards, a comparison can be made between an inferred evidence genotype and a reference, to calculate a LR [31] relative to a population. Separating mixture data into single source-like genotypes can make results easier to explain [33] and simpler to report [42].

## Procedure

### TrueAllele Processing

For each received batch of cases, Cybergenetics processed the.fsa files in the TrueAllele Analyze module to assess data quality. For computing efficiency, EPG peaks below the baseline noise level of 10 RFU were not used (since they do not affect the results). The quality-checked quantitative peak data were then uploaded to a TrueAllele database.

A trained first TrueAllele operator processed a case by downloading from the database the electronic data for all evidence items. The operator examined the EPG signals, and estimated the number of contributors for each evidence item based on the number of peaks observed at each locus. If relevant and available, an assumed reference could be used. (For example, with an intimate sample from a sexual assault, assuming the victim’s genotype as a known contributor to the mixture is forensically meaningful). Appropriate DNA interpretation case questions were uploaded as “requests” from the VUIer to the TrueAllele database for processing.

Following this initial processing, an experienced second TrueAllele operator then reviewed the computer results, and determined whether further analysis would be required. Such additional TrueAllele analyses could entail assuming a different number of contributors, considering DNA degradation, or repeating the question using more computer processing time. When the number of contributors was ambiguous, multiple contributor assumptions were tested; the assumed number of contributors (when there are enough) does not have a major effect on the inferred genotypes or match statistics. Reportable DNA results were replicated in two or more independent computer runs.

### Case Reporting

A reporting scientist examined all the computer results in a case. After careful review of the replicated genotypes, together with the data and mixture weights, a concordant genotype subset was identified. Concordant genotypes had similar probability distributions, mixture weights and Kullback-Leibler (KL) statistics [43]. These properties all measure the inferred genotype, and are independent of any reference comparison or match result. The scientist chose a representative genotype from this concordant set.

In the VUIer program, the TrueAllele scientist indicated the three genotypes (evidence, reference and population) needed to calculate a LR. Virginia’s databases of Black, Caucasian and Hispanic populations were used, and the co-ancestry coefficient was set at 1%. All three LRs were reported; for comparison purposes in this study, we conservatively took the smallest of the three match statistics.

### Match Statistic Collation

Cybergenetics processed the data, and prepared DNA match reports for 72 cases requested by DFS. These cases encompassed 92 items of evidence and 111 comparisons to reference individuals. TrueAllele LR values were collated from these reports. DFS had independently conducted manual mixture calculations on most of the reported TrueAllele matches. These CPI and mCPI match statistics were collected and recorded in a LR format.

A DFS forensic examiner assessed DNA evidence to determine whether a person of interest could be eliminated from the data. This assessment considered the number of contributors, sample type, DNA quantity, potential drop out, and other factors. When the data were inconclusive or the person had been eliminated, no match statistic was calculated.

## Results

We assessed the reliability of DNA mixture interpretation methods through information metrics based on log(LR). The data set comprised 111 computer-inferred evidence genotypes and match statistics; our focus is on the 101 reported matches. We consider in turn how specifically, precisely and sensitively the TrueAllele system performs. Information comparisons with CPI and mCPI methods are possible because formally these manual methods are LRs [28].

Recall that the identification hypothesis H is that a particular individual contributed their DNA to biological evidence. The alternative hypothesis ∼H is that they did not, i.e., that the DNA was left by someone else. Forensic science standardly approximates ∼H with a random man hypothesis that the DNA contributor is an unrelated person selected at random from a genotype population [31].

### TrueAllele Specificity

Specificity measures the extent to which a mixture interpretation method does not misidentify the wrong person. Since identification information is expressed through the log(LR), let X be a real-valued random variable of log(LR) values. We want to understand the TrueAllele distribution of Pr{X = x | ∼H}, the information X conditioned on randomly selected genotypes (that are not contributors to the mixture). The specificity statistic Pr{X>0 | ∼H} then tells us how frequently a positive log(LR) occurs by chance.

Toward this end, we generated ten thousand random genotypes from each of the three Virginia ethnic populations. This generation was done by randomly selecting alleles in proportion to their prevalence in the population database. We compared the 101 matching TrueAllele-inferred evidence genotypes to these random reference genotypes, relative to the appropriate population, to calculate log(LR) values; the co-ancestry coefficient was set to 1%. These values provided a representative log(LR) sampling of over a million nonmatching comparisons for each population.

**Figure 5:**
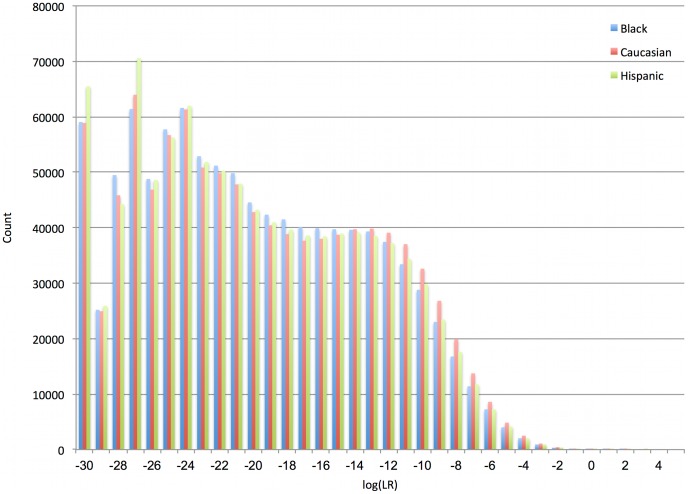
Computer specificity. A histogram shows empirical log(LR) distributions for 101 evidence genotype comparisons relative to 10,000 randomly generated references. There are 1,010,000 data points for each of the three ethnic populations. Note that the negative values are located far to the left of zero.

The resulting empirical Pr{X = x | ∼H} distribution is shown in Figure 5, where the k^th^ bin aggregates the log(LR) values for the interval k ≤ x<k+1. A negative log(LR) value means that a coincidental match is more probable than the evidence matching the reference genotype. TrueAllele’s log(LR) distribution is highly negative, with an average value around –19.5 (Table 6). Thus, for noncontributors, the computer-inferred probability of an evidence genotype is generally much less than the population frequency.

**Table 6:** Specificity results (ban) for TrueAllele mixture interpretation log(LR) values, comparing 101 reported evidence genotypes with 10,000 random genotypes from each of three ethnic populations.

n = 3,030,000	Black	Caucasian	Hispanic
Minimum	−30.000	−30.000	−30.000
Mean	−19.467	−19.217	−19.547
Maximum	2.381	2.726	3.782
Standard deviation	6.543	6.723	6.637
**Tail distribution**	**Black**	**Caucasian**	**Hispanic**
0	39	32	29
1	8	11	9
2	2	1	1
3	0	0	1
log(LR) >0	49	44	40

The average exclusionary LR value was around one over a billion billion. Very few false positives were seen in over three million genotype comparisons.

The specificity value Pr{X>0 | ∼H} was estimated by counting the fraction of positive log(LR) outcomes. For all three ethnic populations, TrueAllele’s false positive rate was less than one in twenty thousand (Table 6, tail distribution). The rate for X>3 was under one in a million, and no false positives were seen beyond that level. The results were essentially the same when reference genotypes were randomly generated using co-ancestry coefficients ranging from 1% to 5% (data not shown).

### TrueAllele Precision

TrueAllele’s genotype model has hundreds of variables. Therefore the (largely continuous) probability model cannot be solved directly by brute force integration or enumeration. Instead, MCMC computing is used to statistically sample from the joint posterior probability distribution, a standard numerical solution for high-dimensional hierarchical models. Such methods exhibit sampling variation between independent computer runs.

**Figure 6:**
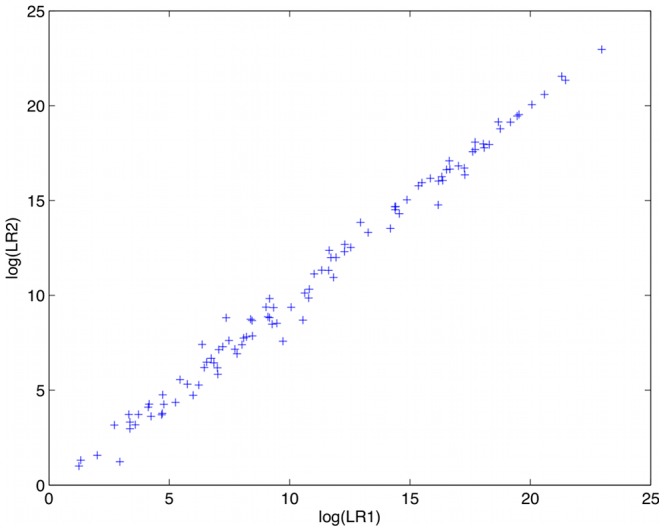
Computer precision. The scatterplot shows log(LR) values for 101 duplicate computer runs on the same evidence. Each point gives the first (x) and second (y) values. The data lie close to the y = x diagonal, which represents exactly replicated results.

Precision describes a method’s reproducibility on the same data. To measure precision, we examined the identification information obtained in duplicate computer runs of the 101 matching genotypes. The observed log(LR) pairs are shown in Figure 6, where the scatterplot shows the points clustering near the y = x diagonal line. Precision can be quantified by calculating within-group standard deviation [7], which is the mean square variation over replicate computations. For the set of genotype matches, we found a precision of 0.305 ban. So, on average, repeated TrueAllele LR values vary by a factor of 2 (10^0.305^) standard deviations.

The log(LR) variation between computer runs was generally greater at medium LR values having logarithms between 5 and 10 (Figure 6). When the LR was small, so were the inter-run deviations. With large LRs, the highly informative genotypes were very reproducible. Statistical tests for heteroscedasticity (Breush-Pagan, White) were not significant (p>0.05).

### TrueAllele Sensitivity

Sensitivity measures the extent to which a mixture interpretation method identifies the correct person. We therefore examine Pr{X = x | H}, the log(LR) distribution conditioned on the identification hypothesis H. In this observational case study, we want reassurance that H is true, so that the reference genotype actually contributed to the mixture evidence.

The preceding specificity results demonstrated that the false positive rate Pr{X>0 | ∼H} of TrueAllele’s mixture interpretation under the noncontributor hypothesis was less than 0.005%. (This is ten times smaller than the highly reliable 0.05% error rate for dual manual review of easily interpreted single-source reference samples [44]). Moreover, beyond small log(LR) levels around 3 ban, no false positives were seen in millions of comparisons. Indeed, in experimental studies based on samples of “known” composition [13], [16], [45], [46], TrueAllele is used to rectify laboratory errors in genotype composition and mixture weight. Since the method’s high specificity assures identification hypothesis H with considerable certainty, we can safely examine the Pr{X = x | H} sensitivity distribution of positive log(LR) values.

**Figure 7:**
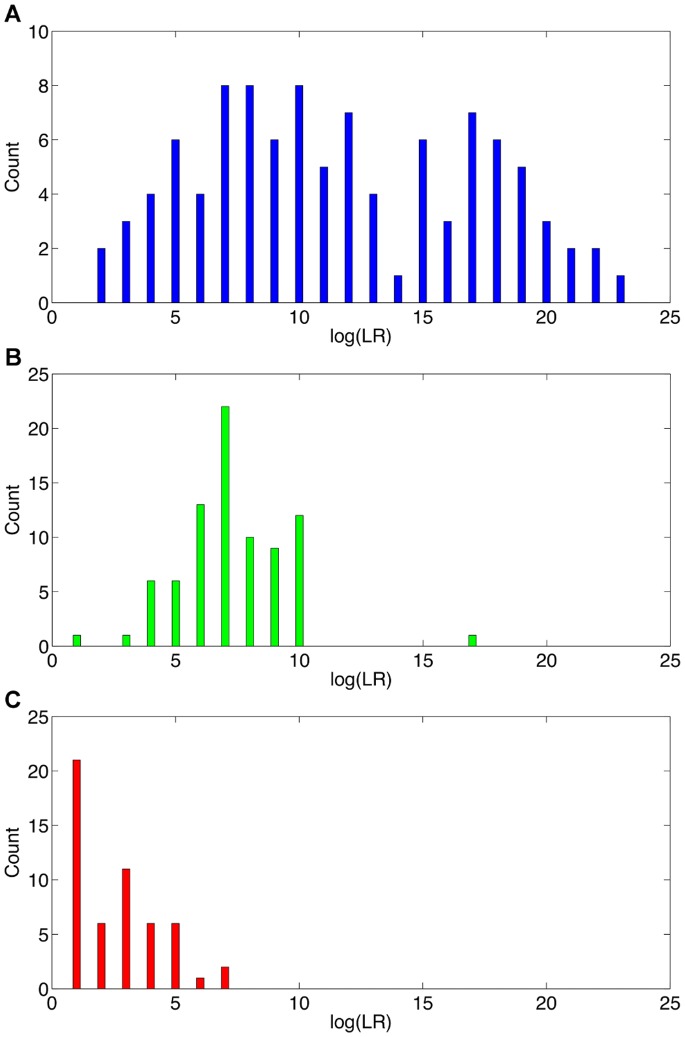
Method sensitivity. Three histograms show the empirical log(LR) distribution for different mixture interpretation methods on the case data. Frequency distribution (**a**) shows TrueAllele inferred genotype match statistics for 101 evidence genotype matches (blue). The (**b**) manual CPI review yielded 81 match statistics (green) that were generally less informative (leftward) and less varied (clustered). The (**c**) 53 mCPI match statistics (red) gave less information and had similar values.

The TrueAllele log(LR) distribution of the 101 reported matches is shown in Figure 7a. For each genotype comparison, we took the smallest of the three ethnic population LR values and used a co-ancestry correction of 1%. The log(LR) values ranged from 1.255 to 22.962, with a mean value of 11.054 ban (Table 7, TrueAllele). As expected, the matching DNA evidence evenly spanned the entire range of positive identification information, from zero to full single-source levels beyond 20 ban. This breadth of scores was also seen in the large standard deviation of 5.421 ban. Of the 101 reported matches, 82 had a DNA statistic exceeding a million, which is a level that people may find persuasive [47].

**Table 7:** The log(LR) DNA match information (ban) for genotype comparisons is shown for three mixture interpretation methods (TrueAllele, CPI and mCPI).

	TrueAllele	CPI	mCPI
Minimum	1.255	0.778	0.301
Median	10.550	6.681	1.857
Mean	11.054	6.825	2.145
Maximum	22.962	16.724	6.447
Standard deviation	5.421	2.217	1.675
N =	111	81	70
Inclusion (≥0)	101	81	53
Persuasive (≥6)	82	54	2
Inconclusive			17

The TrueAllele method preserved more identification information (mean) over a broader range (minimum, maximum) than the two inclusion methods, and produced more inclusions and persuasive match statistics.

More accurate genotype modeling employs a likelihood function that better explains the data, and so tends to produce a higher LR (relative to less accurate modeling) when there is a true match. However, the actual LR value depends on the genotype model, thus some other measure of accuracy is needed. Over a large ensemble of DNA mixtures having randomly distributed mixture weights (Table 5) and DNA amounts, one would expect to observe uniformly distributed identification information. So one measure of accuracy is the degree to which a method’s empirical log(LR) distribution resembles a uniform distribution.

**Figure 8:**
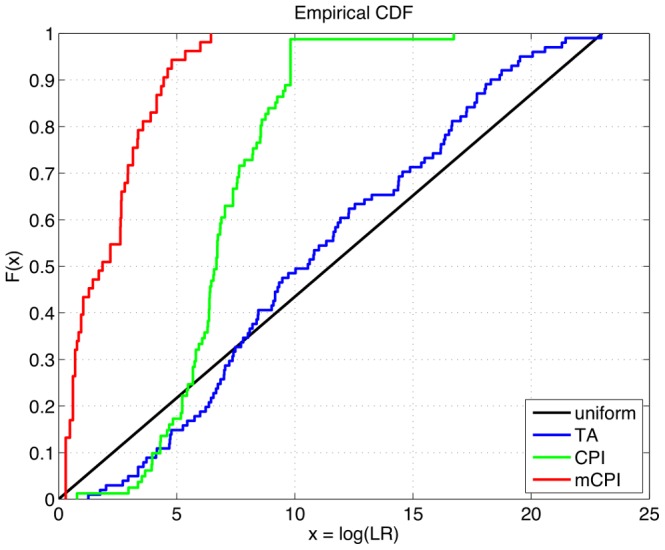
Method comparison. Cumulative empirical log(LR) distributions are shown for uniform probability (black), and for each of the three mixture interpretation methods TrueAllele (blue), CPI (green) and mCPI (red). TrueAllele tracks a uniform distribution over a wide information range, whereas CPI and mCPI do not.

A uniform probability density function (PDF) is a constant horizontal line. TrueAllele’s empirical PDF appears relatively constant across its range of observed log(LR) values (Figure 7a). A better comparison is made using a cumulative distribution function (CDF); for a constant PDF value, the CDF is a straight line moving from 0 up to 1 (Figure 8, black). The computer’s empirical CDF forms a reasonably straight line (Figure 8, blue), similar to the uniform CDF (Figure 8, black). The Kolmogorov-Smirnov (K–S) test can statistically assess whether two probability distributions are the same. With a KS value of 0.1059, the p-value (0.2149>0.05) showed no significant difference between TrueAllele’s empirical log(LR) distribution and a uniform distribution, providing statistical support for the system’s accuracy.

### Threshold Methods

The two threshold-based manual methods produced less informative DNA statistics that were distributed differently than the computer’s 101 genotype modeling positive log(LR) results. On 81 comparisons, the CPI manual method yielded matches with a mean log(CPI) value of 6.825 ban (Table 7, CPI). The mCPI stochastic threshold method gave 53 matches with a 2.145 ban average, and 17 inconclusive results where a match statistic could not be calculated (Table 7, mCPI). Frequency plots of the log(CPI) and log(mCPI) distributions show a pronounced leftward shift for these two match statistics (Figures 7b and 7c, relative to 7a). The match information range narrowed, with standard deviations of 2.217 and 1.675 ban, respectively.

We can again use the Kolmogorov-Smirnov statistic to test the accuracy of these two manual methods. The empirical CDFs of inferred log(LR) values for both CPI (Figure 8, green) and mCPI (Figure 8, red) are seen to deviate from a uniform distribution (Figure 8, black). For CPI, KS = 0.5609 (p = 1.8856×10^–22^), demonstrating a significant difference between CPI’s log(LR) CDF and the uniform distribution. Similarly with mCPI, KS = 0.7352 (p = 1.1316×10^–25^), showing a significant difference between mCPI’s log(LR) CDF and the uniform distribution. The nonuniform clustering of CPI and mCPI log(LR) values (Figures 7b and 7c; Figure 8, green and red), statistically confirmed by the KS tests, does not support the accuracy of threshold methods.

### Comparison of Methods

The numerical differences in average log(LR) between the three interpretation methods were statistically significant (Tables 8–10). TrueAllele preserved the most information, CPI kept less, and mCPI retained the least. These results are not surprising [7]: threshold methods make less use of the data [48], higher thresholds further reduce information, and the study’s case criteria selected for items having low mCPI values. The correlations are also of interest.

**Table 8:** Paired comparisons for positive log(LR) values between TrueAllele (TA) and CPI.

N = 81	TA	CPI	TA – CPI	test	p-value
**Mean**	11.623	6.825	4.798	t = 8.396	1.350×10^–12^
**Median**	10.816	6.681	4.135	W = 3047	6.664×10^–11^
r = 0.2999					
r^2^ = 0.0900					

Significance tests were done for means (Student t) and medians (Wilcoxon signed rank W). Correlation coefficients (r) and coefficient of determinations (r^2^) are shown. TrueAllele was significantly more informative than CPI.

**Table 9:** Paired comparisons for positive log(LR) values between TrueAllele (TA) and mCPI.

N = 53	TA	mCPI	TA – mCPI	test	p-value
**Mean**	12.883	2.145	10.738	t = 15.147	1.040×10^–20^
**Median**	12.537	1.857	10.679	W = 1431	2.386×10^–10^
r = 0.2945					
r^2^ = 0.0867					

Significance tests were done for means (Student t) and medians (Wilcoxon signed rank W). Correlation coefficients (r) and coefficient of determinations (r^2^) are shown. TrueAllele was significantly more informative than mCPI.

**Table 10:** Paired comparisons for positive log(LR) values between CPI and mCPI.

N = 52	CPI	mCPI	CPI – mCPI	test	p-value
**Mean**	7.069	2.180	4.889	t = 17.417	4.082×10^–23^
**Median**	6.720	2.024	4.696	W = 1378	3.497×10^–10^
r = 0.5188					
r^2^ = 0.2692					

Significance tests were done for means (Student t) and medians (Wilcoxon signed rank W). Correlation coefficients (r) and coefficient of determinations (r^2^) are shown. CPI was significantly more informative than mCPI.

The TrueAllele genotype modeling method showed a significant improvement over the older CPI allele inclusion method (Table 8). The mean log(LR) difference was 4.798 (Student t = 8.396, p = 1.350×10^–12^), and the median difference was 4.135 (Wilcoxon sign rank W = 3047, p = 6.664×10^–11^); both differences exceed four orders of magnitude. There is only a weak correlation (r = 0.2999) between the methods, and the small coefficient of determination (r^2^ = 0.0900) leaves over 90% of the variance unexplained. To the extent that TrueAllele quantitative modeling measures identification information, the CPI binary allele inclusion method is measuring something else.

TrueAllele also showed a significant improvement over the newer mCPI allele inclusion approach (Table 9). Here the mean log(LR) difference was 10.738 ban (t = 15.147, p = 1.040×10^–20^), and the median difference was 10.679 ban (W = 1431, p = 2.386×10^–10^). The 10 ban difference is a factor of ten billion in DNA match statistic. The weak correlation (r = 0.2945) and small coefficient of determination (r^2^ = 0.0867) again leaves over 90% of the variance unexplained. Since TrueAllele quantitatively measures identification information, the mCPI stochastic threshold method apparently measures some other data attribute.

Switching from allele inclusion to stochastic thresholds significantly reduced the match statistic (Table 10). The mean log(LR) difference between CPI and mCPI was 4.889 ban (t = 17.417, p = 4.082×10^–23^), and the median difference was 4.696 ban (W = 1378, p = 3.497×10^–10^), which is a match statistic ratio of over ten thousand. There is some correlation (r = 0.5188) between CPI and mCPI, but the small coefficient of determination (r^2^ = 0.2692) does not explain over 70% of the variance. Stochastic thresholds seem to measure inclusion in a different way than does CPI.

These concordant multi-method match results increase confidence in the sensitivity experimental design, where reference genotypes were considered to be present in their respective DNA mixture items. Each of the three interpretation methods works differently, is accepted by courts as reliable criminal evidence, and was calculated independently in the study. In each pairwise comparison, the methods independently agreed on all matches (N>50) and gave positive identification information. These pairwise consensus results were obtained on highly reliable data subsets of the more readily interpretable mixtures.

### TrueAllele Conservatism

Out of 111 TrueAllele genotype comparisons, 10 gave a negative log(LR) value, and so did not produce a positive match result (Table 11, first 10 rows). This often occurred when a reference sample allele was not seen as an STR peak in the evidence data, which could be explained by either exclusion or allele dropout. Dropout at a locus usually yields a negative log(LR) value for that locus, which the computer must tally in its joint match statistic. Other STR features that can confound a match between the evidence data and a reference sample are allele overlap, low peaks, peak imbalance, infeasible mixture combinations and an infeasible mixture pattern.

**Table 11:** Results are shown for ten genotype comparisons where TrueAllele did not report a match, and five others having a small LR value under a thousand.

Interpretation Method			Data Observations					
TrueAllele	CPI	mCPI	allele dropout	allele overlap	low peaks	peak imbalance	infeasible mixture	infeasible pattern
−10.64			3	4	1			1
−6.52			4	3	1			1
−5.05			4	3	1	1		1
−4.87				3		1	1	1
−4.86	3.48		4		1		1	
−3.22	6.04	6.34		2			1	1
−2.99	4.23		2		1		1	1
−2.18			2		1		1	
−1.41	4.08		1		1		1	
−0.67	2.95	0.60	1	2	1			
1.26	3.96		1	4			1	
1.76			1			1	1	
2.01			2	8		1	1	
2.71			2				1	
2.94				8		1		

*Allele dropout* and *allele overlap* record the number of locus occurrences.

*Allele dropout* occurs when a reference allele does not appear at all in the evidence data.

*Allele overlap* occurs when known contributors and the reference share alleles.

*Low peaks*: All had reference-related allele peaks <100 RFU. A 1 indicates peaks <50 RFU.

*Peak imbalance*: a 1 indicates heterozygote imbalance under 60% at reference alleles.

An *infeasible mixture* (1) has an inconsistent mixture weight across loci.

An *infeasible pattern* (1) cannot be constructed quantitatively from contributor genotypes.

Each comparison row gives log(LR) match statistics (ban) for three mixture interpretation methods, and lists observations about how the evidence data interacted with the reference genotype.

There were 5 genotype comparisons where CPI indicated a match, but the computer found no statistical support (Table 11, TrueAllele <0, CPI >0). Laboratory reexamination of these items agreed with the computer’s conclusions. Since the threshold methods did not use peak height information, they supported inclusions whose genotype mixture combinations were incompatible with the quantitative data. Manual mixture interpretation statistics may omit loci that do not demonstrate inclusion, and reported loci can only add positive log(LR) values. The TrueAllele computer, on the other hand, must use all the loci, with negative results at a locus decreasing the total weight of evidence.

Five genotype comparisons gave a small positive TrueAllele log(LR) value of under 3 ban (Table 11, last 5 rows). CPI produced a match statistic in one of these cases, while mCPI provided no statistics. Three of the five items showed considerable allele overlap, which the computer could mathematically resolve better than inclusion methods. There were fewer low peaks and greater peak imbalance in these data, relative to the negative match results. The last column shows that TrueAllele can distinguish between quantitatively feasible and infeasible mixture patterns, while CPI and mCPI may not.

## Discussion

Modern criminal justice requires rapid and reliable processing of DNA evidence. Reliability is the basis of admissible evidence, and entails sensitivity, specificity and precision. However, when confronted with complex mixtures or touch DNA, manual review can become a challenging task. Since such mixtures may constitute the bulk of biological evidence found in serious crimes such as sexual assault or homicide, effective interpretation of these data is needed.

In this casework study, the newly adopted mCPI stochastic threshold method produced results in 53 of 70 DNA match comparisons, finding an average match statistic of 140 (Table 7, mCPI, as 10^2.145^). The previously used CPI threshold method had greater sensitivity in 81 inclusions on this data set, for an average match statistic of 6.68 million. TrueAllele computer interpretation provided 101 match statistics, with an average LR of 113 billion. The genotype modeling reported on more of the evidence than did threshold methods, and preserved more DNA identification information.

TrueAllele mixture interpretation does not always increase a DNA match statistic. In this study, the computer’s statistics were lower than the corresponding human CPI values in 15 reported matches [49]. Moreover, the computer found no statistical support for a match in 10 cases, including 5 where CPI gave an inclusionary match statistic. While the system does find more matches and computes stronger statistics on average, it examines DNA evidence objectively without introducing bias that may favor the prosecution or defense.

In addition to increased average sensitivity, TrueAllele also maintains excellent specificity. The computer’s LR can quantify negative match information, unlike manual interpretation methods that are restricted to positive (logarithmic) values. We examined several million genotype comparisons between computer-inferred evidence and randomly generated references, and found a false positive rate of under 0.005%. The negative match information in this simulation experiment had a log(LR) averaging around −19.5 ban.

TrueAllele calculates DNA match statistics with precision. Replicate computer runs on the same evidence data showed a within-group log(LR) standard deviation of 0.305 ban. Thus, independent runs on the same evidence item gave statistically similar DNA match statistics that were usually within an order of magnitude.

To assess accuracy, (logarithmic) match statistic distributions were examined. Across an entire ensemble of matches, the random sampling inherent in casework should produce a uniform log(LR) distribution. TrueAllele’s match distribution was statistically uniform, which lends support to the overall accuracy of its LR values. However, the manual methods each clustered around their average match value, and so did not exhibit a uniform distribution that would support their accuracy.

DNA, whether single source or complex mixture, can provide evidence that implicates criminals and exonerates the innocent. Current manual review of DNA mixture data applies thresholds that can discard valuable data and understate the evidential import of the identification information. As demonstrated in this casework comparison study, TrueAllele computer interpretation more effectively preserves DNA evidence and match information, relative to CPI and mCPI methods that use thresholds. Both prosecutors and defense attorneys may benefit from use of this validated computer technology to review complex DNA mixture evidence.
